# Frequency of flash glucose monitoring and glucose metrics: real-world observational data from Saudi Arabia

**DOI:** 10.1186/s13098-022-00831-y

**Published:** 2022-05-03

**Authors:** Mohammad Y. Al-Harbi, Abdulhameed Albunyan, Ahmed Alnahari, Kalvin Kao, Laura Brandner, Manal El Jammal, Timothy C. Dunn

**Affiliations:** 1 Therapeutic Services Deputyship, Ministry of Health, Riyadh, Kingdom of Saudi Arabia; 2 Ministry of Health, Riyadh, Kingdom of Saudi Arabia; 3 Clinical Affairs, Abbott Diabetes Care, Alameda, CA USA; 4 Scientific & Medical Affairs, Abbott Diabetes Care, Alameda, CA USA

**Keywords:** Flash glucose monitoring, Real-world data, FreeStyle Libre^®^

## Abstract

**Background:**

This real-world data study analyzed glucose metrics from FreeStyle Libre^®^ flash glucose monitoring in relation to scanning frequency, time in range (TIR) and estimated A1c (eA1c) in Saudi Arabia.

**Methods:**

Anonymized reader data were analyzed according to scanning frequency quartiles, eA1c categories (<7%,≥7%‒≤9% or>9%) and TIR categories (<50%,≥50%‒≤70% or>70%). Sensors, grouped by reader, were required to have≥120 h of operation. Differences in scanning frequency, eA1c, TIR, time in hypoglycemia and hyperglycemia, and glucose variability (standard deviation [SD] and coefficient of variation [CV]) were analyzed between groups.

**Results:**

6097 readers, 35,747 sensors, and 40 million automatic glucose measurements were analyzed. Patients in the highest scanning frequency quartile (Q4, mean 32.0 scans/day) had lower eA1c (8.47%), greater TIR (46.4%) and lower glucose variation (SD 75.0 mg/dL, CV 38.2%) compared to the lowest quartile (Q1, mean 5.2 scans/day; eA1c 9.77%, TIR 32.8%, SD 94.9 mg/dL, CV 41.3%). Lower eA1c and higher TIR were associated with greater scanning frequency, lower glucose variability and less time in hyperglycemia.

**Conclusions:**

Higher scanning frequency in flash glucose users from Saudi Arabia is associated with lower eA1c, higher TIR, lower glucose variability and less time in hypoglycemia or hyperglycemia.

**Supplementary Information:**

The online version contains supplementary material available at 10.1186/s13098-022-00831-y.

## Background

Diabetes mellitus is a chronic metabolic disorder characterized by high blood glucose levels resulting from impaired insulin production or response [1]. The global prevalence of diabetes is high and rising fast, with 463 million individuals suffering from diabetes in 2019 and projected estimates of 700 million individuals with diabetes by 2045 [2]. In Saudi Arabia, a country with a population of approximately 30 million, diabetes is a major public health concern, with more than 1.7 million individuals aged 15 years and older suffering from diabetes and a further 751,684 individuals with undiagnosed diabetes in 2014 [3]. Complications of poorly managed diabetes include microvascular complications; (neuropathy, nephropathy, and retinopathy), as well as macrovascular disease such as stroke, coronary artery disease, and peripheral arterial disease resulting from hyperglycemia [4]. Hypoglycemia can be associated with seizures, coma, cardiac issues as well as cognitive impairment [5].

Glycated hemoglobin (HbA1c) is used to measure glycemic control and correlates with mean glucose levels [6]. In addition to the measurement of HbA1c, self-monitoring of blood glucose levels allows individuals with diabetes to monitor their own glucose levels in relation to treatment response and glycemic targets [6]. Intensive treatment of insulin-dependent diabetes patients—which includes regular insulin treatment, self-monitoring of blood glucose levels, and regular measurement of HbA1c—has been shown to reduce the progression of complications associated with diabetes [7].

Advances in glucose monitoring have led to the development of continuous glucose monitoring devices, which can track glucose levels continuously throughout the day. Continuous glucose monitoring has been shown to reduce hypoglycemia events, improve quality of life, and improve HbA1c levels [8].

Increased frequency of self-monitoring of blood glucose has been shown to be associated with reduced HbA1c levels in type 1 diabetes patients of all ages [9]. Randomized control trials have shown that frequent flash glucose monitoring can reduce time spent in hypoglycemia for type 1 diabetes patients with well-controlled diabetes [10], as well as in type 2 diabetes patients on intensive insulin therapy [11]. While previous real-world data studies have shown greater flash glucose monitoring frequency to be associated with improved glycemic markers, including increased time in range (TIR), reduced time in hypoglycemia and hyperglycemia, and reduced glucose variability in European populations [12–14], none have assessed this association within the Saudi Arabian population.

TIR, which is a standardized continuous glucose monitoring metric, is associated with a reduction in the risk of microvascular complications [15, 16]. This metric is thought to overcome limitations associated with the reliance on HbA1c alone to assess glycemic control and provide more information about acute glycemic events [17, 18]. The relationship between TIR, eA1c and glycemic variability has previously been studied in a Chinese population of people with type 2 diabetes and demonstrated the complexity of determining TIR targets for individual patients [19].

The aim of this real-world data study was to analyze glucose metrics from flash glucose monitoring in relation to scanning frequency, time in range and eA1c in Saudi Arabia.

## Methods

### Sensors and readers

We used The FreeStyle Libre^®^ (Abbott Diabetes Care, Witney, UK) flash glucose monitoring system which is a sensor-based continuous glucose monitor that measures glucose levels in the interstitial fluid every minute, and automatically saves a reading every 15 min [20]. A reader which is scanned over the sensor will display up to 8 h of historical glucose measurements, as well as current glucose and glucose trends [20]. This device does not require calibration and has a 14-day sensor life [12]. Anonymized data from all flash glucose monitoring glucose readers from Saudi Arabia was uploaded and analyzed for the period of October 2015 to June 2020. Sensors were grouped by reader and were required to have ≥ 120 h of operation.

### Scanning

Each reader’s daily scanning frequency was calculated by dividing the total number of scans from all its associated sensors by the total wear duration (in days) of all its associated sensors.

### Glycemic measurements

Glycemic measurements calculated from the sensor data included eA1c (%), TIR (% time glucose levels were between 70 and 180 mg/dL), time above range (% time glucose levels were > 180 mg/dL and > 250 mg/dL), time below range (% time glucose levels were < 70 mg/dL and < 54 mg/dL), and glucose variability metrics of standard deviation (SD; mg/dL) and coefficient of variation (CV; %).

### Statistical analysis

For analysis of the relationship between scanning frequency and glucose metrics, readers were rank ordered by scanning frequency into quartiles, with Q1 representing the lowest scanning frequency quartile and Q4 representing the highest scanning frequency quartile. For analysis of the relationship between eA1c and glucose metrics, eA1c was categorized as < 7%, ≥ 7% and ≤ 9% or > 9%. For analysis of the relationship between TIR and glucose metrics, TIR was categorized as < 50%, ≥ 50% and ≤ 70% or > 70%.

Mean values with 95% confidence intervals were calculated for eA1c, TIR, time in hyperglycemia ranges, and glucose variability metrics, for each scanning frequency quartile, eA1c category or TIR category. For time in hypoglycemia ranges, median values with 95% bootstrapped confidence intervals of the median were calculated instead of means, due to the high number of zeros and skew in the distribution of time in hypoglycemia. Analysis of variance was used to assess differences in mean glucose metrics between scanning frequency quartiles, eA1c category or TIR category. Differences in median time in hypoglycemia between scanning frequency quartiles were assessed by a comparison of their calculated confidence intervals. The database was analyzed by structured query language routines and the Python programming language (www.python.org, version 3.7.3), and further summarized by KNIME (http://www.knime.org, version 4.1.3). Comparisons were considered significantly different at the p < 0.05 level.

A supplementary analysis of the relationship between the standardized metric Glucose Management Index (GMI) [21] and other glucose metrics was performed. GMI was categorized as < 7%, ≥ 7% and ≤ 9% or > 9% and analyzed as described above for scanning frequency quartile, eA1c and TIR categories.

## Results

### Automatic glucose measurements

The dataset from Saudi Arabia contained information from a total of 6097 readers, 35,747 sensors, and 40 million automatic glucose measurements.

### Relationship between scanning frequency and glucose metrics

**Fig. 1:**
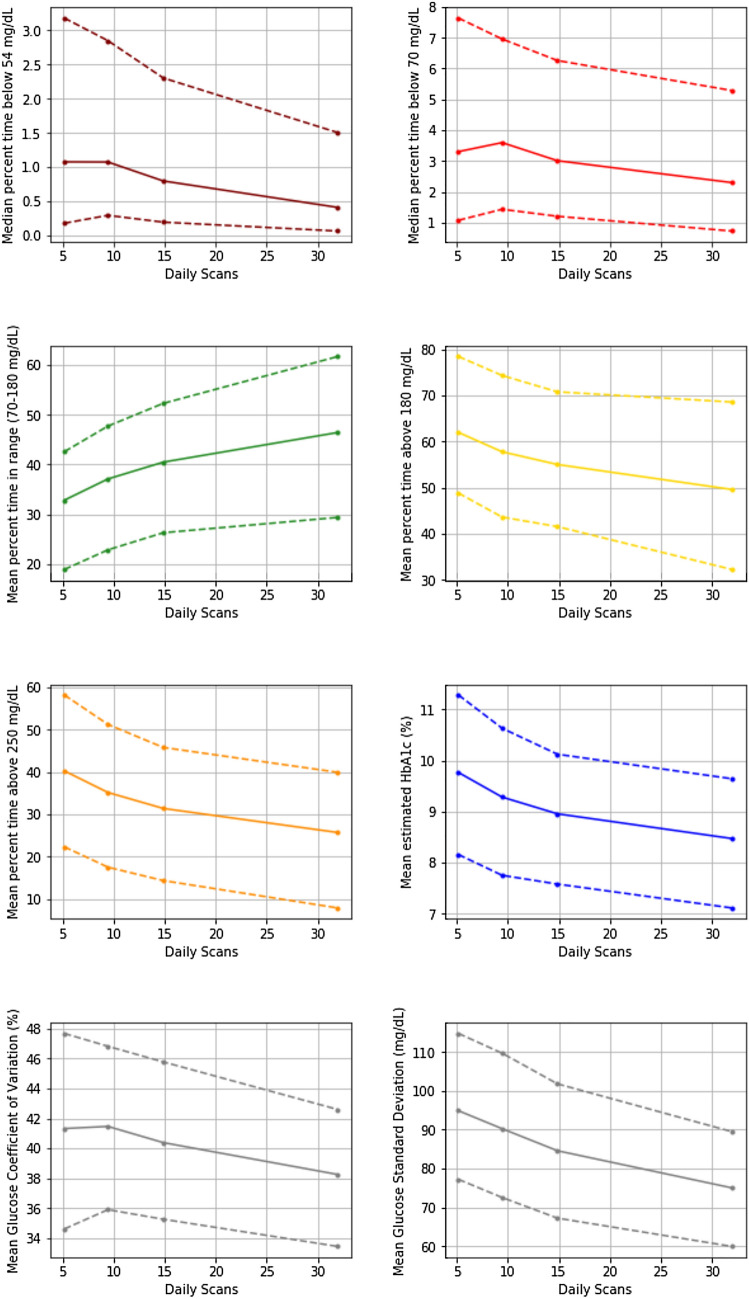
Relationship between scanning frequency and glucose metrics Mean values are connected by solid lines, 75th and 25th percentile values are connected by dashed lines. For percent time below 54 and 70 mg/dL, median values are connected by solid lines.

Glucose metrics according to each scanning frequency quartile are shown in Table 1 and Fig. 1. Greater scanning frequency was associated with lower mean eA1c, greater mean TIR and lower glucose variability. Greater scanning frequency was also associated with lower time in hypoglycemia as well as lower time in hyperglycemia.

**Table 1:** Glucose metrics by scanning frequency quartile

	Q1	Q2	Q3	Q4	p-value
Number of readers	1523	1524	1524	1526	
Daily scans	5.2 (5.10‒5.23)	9.4 (9.38‒9.51)	14.9 (14.76‒14.97)	32.0 (31.21‒32.71)	< 0.0001
eA1c, %	9.77 (9.65‒9.88)	9.28 (9.18‒9.39)	8.96 (8.86‒9.05)	8.47 (8.38‒8.57)	< 0.0001
Time below range					
< 54 mg/dL, %*	1.08 (0.92‒1.18)	1.07 (1.00‒1.22)	0.80 (0.71‒0.89)	0.41 (0.36‒0.45)	< 0.05
< 70 mg/dL, %*	3.31 (3.00‒3.60)	3.60 (3.40‒3.88)	3.01 (2.77‒3.25)	2.31 (2.03‒2.51)	< 0.05
TIR					
≥ 70 and ≤ 180 mg/dL, %	32.8 (31.9‒33.8)	37.1 (36.1‒38.1)	40.5 (39.5‒41.5)	46.4 (45.4‒47.5)	< 0.0001
Time above range					
> 180 mg/dL, %	62.0 (60.9‒63.1)	57.8 (56.7‒58.9)	55.0 (53.9‒56.1)	49.6 (48.4‒50.8)	< 0.0001
> 250 mg/dL, %	40.3 (39.1‒41.4)	35.2 (34.1‒36.3)	31.4 (30.4‒32.5)	25.7 (24.7‒26.8)	< 0.0001
Glucose variability					
Glucose SD, mg/dL	94.9 (93.5‒96.2)	90.2 (88.9‒91.6)	84.6 (83.3‒85.8)	75.0 (73.8‒76.2)	< 0.0001
Glucose CV, %	41.3 (40.8‒41.8)	41.5 (41.0‒41.9)	40.4 (40.0‒40.8)	38.2 (37.9‒38.6)	< 0.0001

Values are presented as mean (95% confidence interval) unless specified

*CV* coefficient of variation, *eA1c* estimated A1c, *SD* standard deviation, *TIR* time in range

*Median (95% bias-corrected and accelerated bootstrap confidence intervals) presented for time below 54 and 70 mg/dL

### Relationship between eA1c and glucose metrics

Glucose metrics according to eA1c category are shown in Table 2. Lower eA1c was associated with greater mean daily scans, greater TIR and lower glucose variability. Lower eA1c was also associated with lower time in hyperglycemia but slightly higher time in hypoglycemia. Similar results were observed for GMI (data provided in Additional file 1).

**Table 2:** Glucose metrics by eA1c group

	eA1c < 7%	eA1c ≥ 7% & ≤ 9%	eA1c > 9%	p-value
Number of readers	924	2231	2942	
eA1c, %	6.22 (6.17–6.26)	8.04 (8.02–8.06)	10.85 (10.8–10.9)	< 0.0001
Daily scans	19.6 (18.5–20.6)	16.5 (15.9–17.0)	13.2 (12.8–13.6)	< 0.0001
Time below range				
< 54 mg/dL, %*	1.62 (1.33–1.90)	1.25 (1.13–1.37)	0.47 (0.42–0.51)	< 0.05
< 70 mg/dL, %*	7.55 (6.72–8.21)	4.31 (4.07–4.61)	1.76 (1.65–1.89)	< 0.05
TIR				
≥ 70 and ≤ 180 mg/dL, %	73.0 (72.1–73.9)	47.1 (46.7–47.5)	22.6 (22.3–22.9)	< 0.0001
Time above range				
> 180 mg/dL, %	17.6 (17.0–18.3)	47.4 (47.0–47.8)	74.8 (74.4–75.1)	< 0.0001
> 250 mg/dL, %	3.8 (3.6–4.1)	20.0 (19.6–20.3)	52.4 (51.8–52.9)	< 0.0001
Glucose variability				
Glucose SD, mg/dL	50.6(49.4–51.7)	77.8 (77.1–78.5)	103.7 (103.0–104.4)	< 0.0001
Glucose CV, %	37. 7(37.0–38.4)	42.3 (41.9–42.6)	39.7 (39.4–40.0)	< 0.0001

Values are presented as mean (95% confidence interval) unless specified

*CV* coefficient of variation, *eA1c* estimated A1c, *SD* standard deviation, *TIR* time in range

*Median (95% bias-corrected and accelerated bootstrap confidence intervals) presented for time below 54 and 70 mg/dL

### Relationship between TIR and glucose metrics

Glucose metrics according to TIR category are shown in Table 3. Higher TIR was associated with greater mean daily scans, lower eA1c and lower glucose variability. Higher TIR was also associated with less time in hyperglycemia.

**Table 3:** Glucose metrics by TIR group

	TIR < 50%	TIR ≥ 50% & ≤ 70%	TIR > 70%	p-value
Number of readers	4426	1100	571	
TIR	29.0 (28.6–29.3)	58.3 (57.9–58.6)	81.8 (81.1–82.5)	< 0.0001
Daily scans	13.6 (13.3–13.9)	19.7 (18.8–20.6)	20.6 (19.2–22.1)	< 0.0001
eA1c, %	9.99 (9.93–10.04)	7.22 (7.18–7.25)	6.07 (6.01–6.12)	< 0.0001
Time below range				
< 54 mg/dL, %*	0.75 (0.69–0.79)	1.4 (1.21–1.61)	0.48 (0.41–0.55)	< 0.05
< 70 mg/dL, %*	2.53 (2.41–2.66)	5.14 (4.84–5.63)	3.57 (3.09–3.96)	< 0.05
Time above range				
> 180 mg/dL, %	66.9 (66.5–67.4)	34.9 (34.4–35.4)	12.8 (12.1–13.5)	< 0.0001
> 250 mg/dL, %	42.8 (42.3–43.4)	10.5 (10.3–10.8)	1.7 (1.6–1.9)	< 0.0001
Glucose variability				
Glucose SD, mg/dL	97.2 (96.6–97.8)	65.6 (65.0–66.3)	40.4 (39.4–41.4)	< 0.0001
Glucose CV, %	41.3 (41.1–41.6)	41.2 (40.8–41.6)	31.3 (30.7–31.9)	< 0.0001

Values are presented as mean (95% confidence interval) unless specified

*CV*. coefficient of variation, *eA1c* estimated A1c, *SD* standard deviation, *TIR* time in range

*Median (95% bias-corrected and accelerated bootstrap confidence intervals) presented for time below 54 and 70 mg/dL

## Discussion

The results of this real-world data study demonstrate the benefits of greater scanning frequency in flash glucose monitoring users in Saudi Arabia. Higher scanning frequency users demonstrated significantly higher TIR, lower eA1c, lower time in hypoglycemia and hyperglycemia, and lower glucose variability compared with lower scanning frequency users. Furthermore, users who spent more time in range had more favorable glycemic outcomes in terms of eA1c, glucose variability and time above range and had greater scanning frequency compared with users who spent less time in range. Similarly, users with lower eA1c had better glycemic markers in terms of TIR, glucose variability and time above range and had greater scanning frequency compared with users with higher eA1c. Analysis of real-world data demonstrates that higher scanning frequency, lower eA1c and higher TIR in flash glucose monitoring users from Saudi Arabia is associated with improved glycemic markers.

Previous real-world data studies have similarly associated lower eA1c with greater scanning frequency in flash glucose monitoring users. In a Spanish study, eA1c was significantly lower for the highest scanning frequency group (6.9%) than it was for the lowest scanning frequency group (8.0%; p < 0.001) [13]. Furthermore, in a European study, eA1c was higher for the lowest scanning frequency group (8.0%) compared with the highest scanning frequency group (6.7%; p < 0.001) [12]. In a study in Italy, eA1c was significantly higher in the quintile with the lowest number of daily glucose readings (8.4%) versus the quintile with the highest number of daily glucose readings (6.9%; p < 0.001) [22]. Prospective observational studies have reported a similar relationship between daily scan frequency and HbA1c [23–25], with two of the studies reporting a strong correlation between daily scanning frequency and HbA1c or eA1c [24, 25]. A national study in Belgium reported a logarithmic relationship between daily scans and HbA1c [23]; this fit function agrees well with the real-world data reported here. The relative decreases in HbA1c and eA1c are just over 13% in both Belgium [23] and Saudi Arabia across the scan frequencies of 5.2 to 32 per day.

Furthermore, comparable differences in time spent in hypoglycemia and hyperglycemia with greater scanning frequency with flash glucose monitoring have also been observed in other real-world data studies. In a longitudinal analysis over a 6-month period, Jangam et al.demonstrated a greater reduction in hyperglycemia in those individuals with greater scanning frequency (− 0.81 h/day, 14.2% relative reduction) compared with individuals with lower scanning frequency (+0.16 h/day, 2.4% relative increase) [14]. In addition, Gomez-Peralta et al. demonstrated significantly lower time spent below 70 mg/dL (2.1%), below 54 mg/dL (5.9%), and above 180 mg/dL (28.9%) in the highest scanning frequency group compared with the lowest scanning frequency group (3.2%, 6.9%, and 45.4%, respectively) [13]. Dunn et al.demonstrated that higher scanning frequency was associated with reduced time below 3.9, 3.1 and 2.5 mmol/L (70.2, 55.8 and 45.0 mg/dL) by 15%, 40%, and 49%, respectively, compared with lower scanning frequency (p < 0.001 for all groups). Furthermore, time above 10.0 mmol/L (180 mg/dL) decreased by 44% for the higher scanning frequency group (p < 0.001) [12].

In addition, studies have associated greater time spent in range with greater scanning frequency with flash glucose monitoring, with a TIR of 15.6 h/day for higher scanning frequencies versus 11.5 h/day for lower scanning frequencies in the aforementioned Spanish real-world data study [13]. Furthermore, in a European study, TIR was shown to be higher for greater scanning frequencies (12.0 h/day) versus lower scanning frequencies (16.8 h/day, p < 0.001) [12]. In an observational study in Japan, scanning frequency was positively correlated with TIR (r = 0.719, P < 0.0001) [25]. Finally, a retrospective study of patients in Italy demonstrated a significant trend of greater TIR with increasing daily interstitial glucose readings [22].

In terms of glycemic variability, the same Spanish real-world data study associated lower SD and CV with greater scanning frequency with flash glucose monitoring. Glucose SD and CV were 28.7% (p < 0.001) and 13.5% (p < 0.001) lower, respectively, in the higher frequency scanning group than they were in the lower scanning frequency group [13]. A study of people with type 2 diabetes in China has shown a strong correlation between eA1c and TIR, which is influenced by glycemic variability in continuous glucose monitoring users, demonstrating that all these factors need to be accounted for when deciding on a clinical target for each individual patient [19].

This study is the first real-world data study that assesses the association between flash glucose monitoring scanning frequency, time in range and eA1c and glucose metrics in Saudi Arabia. The results confirm findings from previous real-world studies in a new setting. The study enabled the collection of a large sample size in a real-life user setting. However, the limitations to this study include the lack of additional patient information such as patient demographics, diabetes duration, diabetes complications, therapies and self-management information, which may be contributing factors to scanning frequency or glucose metrics. There is the potential for selection bias in this study resulting from the types of individuals using the flash glucose monitoring device, with the potential for those using the device having high levels of motivation for improvement of glycemic indices. Finally, this study did not assess the effect of scanning frequency, time in range and eA1c on glucose metrics over time; there may be the potential for changes in such parameters over the course of flash glucose monitoring use, therefore this may warrant further investigation.

## Conclusions

This study is the first real-world data study that assesses the association between flash glucose monitoring scanning frequency, time in range and eA1c and glucose metrics in Saudi Arabia. Higher scanning frequency in flash glucose users from Saudi Arabia is associated with lower eA1c, higher TIR, lower glucose variability and less time in hypoglycemia or hyperglycemia. The data demonstrates that higher scanning frequency, lower eA1c and higher TIR in flash glucose monitoring users from Saudi Arabia is associated with improved glycemic markers and these results demonstrate the clinical benefit of frequent blood glucose monitoring on glycemic control.

## Supplementary Information

**Additional file1:** Glucose metrics by GMI category

## Data Availability

Data are available upon reasonable request.

## Abbreviations

CV: Coefficient of variation
eA1c: Estimated A1c
GMI: Glucose Management Index
HbA1c: Glycated hemoglobin
SD: Standard deviation
TIR: Time in range

## References

[1] American Diabetes Association (2020). 2. Classification and diagnosis of diabetes: standards of medical care in diabetes-2020. Diabetes Care.

[2] Saeedi P (2019). Global and regional diabetes prevalence estimates for 2019 and projections for 2030 and 2045: results from the International Diabetes Federation Diabetes Atlas, 9(th) edition. Diabetes Res Clin Pract.

[3] Mokdad AH (2015). Cost of diabetes in the Kingdom of Saudi Arabia, 2014. J Diabetes Metab.

[4] Fowler MJ (2008). Microvascular and macrovascular complications of diabetes. Clinical Diabetes.

[5] Frier BM (2014). Hypoglycaemia in diabetes mellitus: epidemiology and clinical implications. Nat Rev Endocrinol.

[6] American Diabetes Association (2020). Glycemic targets: standards of medical care in diabetes-2020. Diabetes Care.

[7] et al., Diabetes Control Complications Trial Research Group (1993). The effect of intensive treatment of diabetes on the development and progression of long-term complications in insulin—dependent diabetes mellitus. N Engl J Med.

[8] Adolfsson P (2018). Hypoglycaemia remains the key obstacle to optimal glycaemic control—continuous glucose monitoring is the solution. Eur Endocrinol.

[9] Miller KM (2013). Evidence of a strong association between frequency of self—monitoring of blood glucose and hemoglobin A1c levels in T1D exchange clinic registry participants. Diabetes Care.

[10] Bolinder J (2016). Novel glucose—sensing technology and hypoglycaemia in type 1 diabetes: a multicentre, non-masked, randomised controlled trial. Lancet.

[11] Haak T (2017). Flash glucose-sensing technology as a replacement for blood glucose monitoring for the management of insulin—treated type 2 diabetes: a multicenter. Diabetes Ther.

[12] Dunn TC (2018). Real—world flash glucose monitoring patterns and associations between self-monitoring frequency and glycaemic measures: a European analysis of over 60 million glucose tests. Diabetes Res Clin Pract.

[13] Gomez-Peralta F (2020). Flash glucose monitoring reduces glycemic variability and hypoglycemia: real—world data from Spain. BMJ Open Diabetes Res Care.

[14] Jangam S (2019). Flash glucose monitoring improves glycemia in higher risk patients: a longitudinal, observational study under real—life settings. BMJ Open Diabetes Res Care.

[15] Beck RW (2019). Validation of time in range as an outcome measure for diabetes clinical trials. Diabetes Care.

[16] Lu J (2018). Association of time in range, as assessed by continuous glucose monitoring, with diabetic retinopathy in type 2 diabetes. Diabetes Care.

[17] Danne T (2017). International consensus on use of continuous glucose monitoring. Diabetes Care.

[18] Battelino T (2019). Clinical targets for continuous glucose monitoring data interpretation: recommendations from the international consensus on time in range. Diabetes Care.

[19] Lu J (2020). Glycemic variability modifies the relationship between time in range and hemoglobin A1c estimated from continuous glucose monitoring: a preliminary study. Diabetes Res Clin Pract.

[20] Blum A (2018). Freestyle libre glucose monitoring system. Clin Diabetes.

[21] Bergenstal RM (2018). Glucose management indicator (GMI): a new term for estimating A1C from continuous glucose monitoring. Diabetes Care.

[22] Laurenzi A (2020). Frequency of flash glucose monitoring readings, hemoglobin A1c and time in range: a real life study in adults with type 1 diabetes. Acta Diabetol.

[23] Charleer S (2020). Quality of life and glucose control after 1 year of nationwide reimbursement of intermittently scanned continuous glucose monitoring in adults living with type 1 diabetes (FUTURE): a prospective observational real—world cohort study. Diabetes Care.

[24] Tyndall V (2019). Marked improvement in HbA1c following commencement of flash glucose monitoring in people with type 1 diabetes. Diabetologia.

[25] Suzuki J (2021). Association between scanning frequency of flash glucose monitoring and continuous glucose monitoring—derived glycemic makers in children and adolescents with type 1 diabetes. Pediatr Int.

