# Supplementary material for: Frequency of flash glucose monitoring and glucose metrics: real-world observational data from Saudi Arabia
Source: Diabetol Metab Syndr. 2022 May 3;14:66. doi: 10.1186/s13098-022-00831-y (PMC9063302; doi:10.1186/s13098-022-00831-y)
Supplement: Supplementary file 1 — Additional file1: Glucose metrics by GMI category [file 13098_2022_831_MOESM1_ESM.docx]

**Supplementary File 1.** Glucose metrics by GMI category

|  | GMI <7% | GMI ≥7% & ≤9% | GMI >9% | p-value |
| --- | --- | --- | --- | --- |
| Number of readers | 926 | 3211 | 1960 |  |
| GMI, % | 6.51  (6.48-6.55) | 8.01  (7.99-8.04) | 10.11  (10.07-10.15) | <0.0001 |
| eA1c, % | 6.22  (6.18-6.26) | 8.47  (8.44-8.50) | 11.56  (11.50-11.62) | <0.0001 |
| Daily scans | 19.6  (18.5-20.6) | 15.9  (15.5-16.4) | 12.5  (12.0-12.9) | <0.0001 |
| Time below range |  |  |  |  |
| <54 mg/dL, %* | 1.62  (1.33-1.91) | 1.09  (1.02-1.18) | 0.34  (0.29-0.38) | <0.05** |
| <70 mg/dL, %* | 7.55  (6.75-8.24) | 3.81  (3.61-4.01) | 1.36  (1.24-1.45) | <0.05** |
| TIR |  |  |  |  |
| ≥70 and ≤180 mg/dL, % | 73.0  (72.1-73.9) | 42.4  (42.0-42.7) | 18.1  (17.8-18.4) | <0.0001 |
| Time above range |  |  |  |  |
| >180 mg/dL, % | 17.6  (17.0-18.3) | 52.7  (52.3-53.1) | 79.8  (79.4-80.2) | <0.0001 |
| >250 mg/dL, % | 3.8  (3.6-4.1) | 25.3  (25.0-25.7) | 59.8  (59.3-60.4) | <0.0001 |
| Glucose variability |  |  |  |  |
| Glucose SD, mg/dL | 50.6  (49.4-51.7) | 83.0  (82.4-83.6) | 108.2  (107.4-109.0) | <0.0001 |
| Glucose CV, % | 37.7  (37.0-38.4) | 42.3  (42.0-42.5) | 38.5  (38.1-38.8) | <0.0001 |

CV, coefficient of variation; eA1c, estimated A1c; SD, standard deviation; TIR, time in range. Values are presented as mean (95% confidence interval) unless specified. *Median (95% bias-corrected and accelerated bootstrap confidence intervals) values presented for time below 54 and 70 mg/dL.
